# Supplementary material for: Modeling the dynamics of mouse iron body distribution: hepcidin is necessary but not sufficient
Source: BMC Syst Biol. 2017 May 18;11:57. doi: 10.1186/s12918-017-0431-3 (PMC5437513; doi:10.1186/s12918-017-0431-3)
Supplement: Additional file 1: — Additional figures, tables, and mathemtical model equations. (PDF 943 kb) [file 12918_2017_431_MOESM1_ESM.pdf]

Modeling the dynamics of mouse iron body distribution: hepcidin is necessary but not sufficient

Jignesh H. Parmar, Grey Davis, Hope Shevchuk and Pedro Mendes

Supplementary Data

Model Process Diagrams

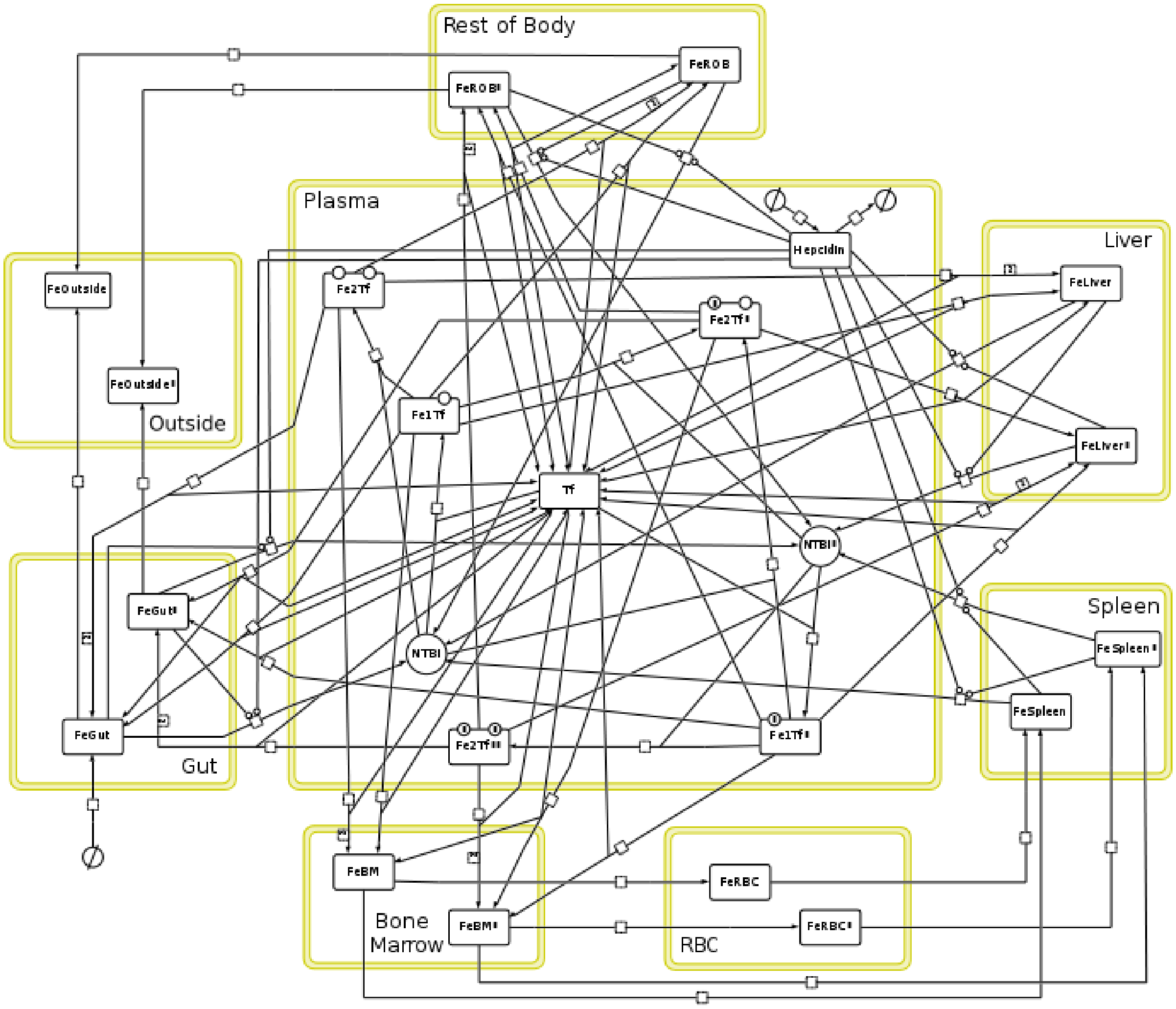

Figure S1. Process description diagram of the complete model including radioactive iron species, according to the systems biology graphical notation (SBGN [23]). The symbol '*' marks the radioactive species.

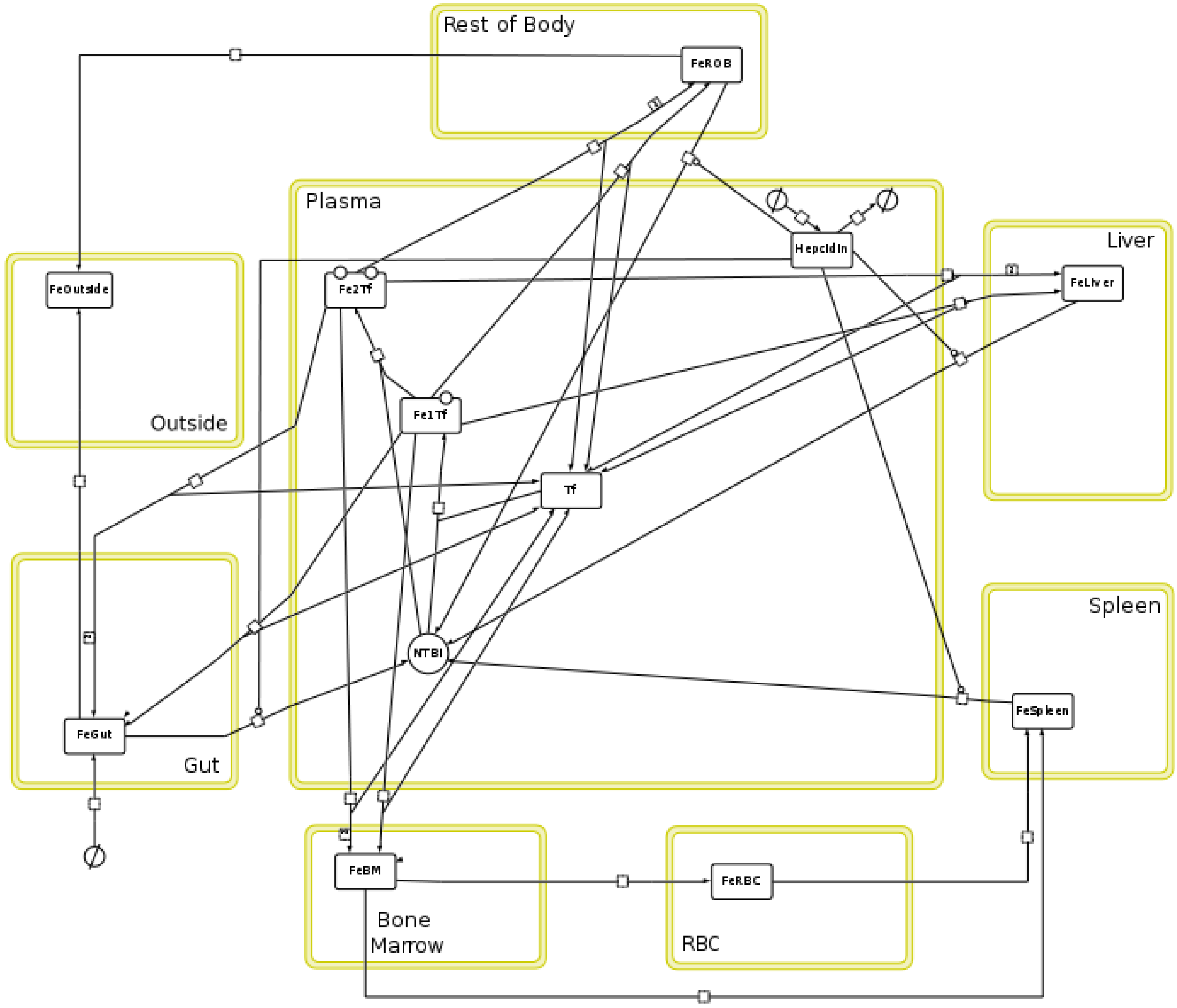

Figure S2. Process description diagram of the model without radioactive iron species, according to the systems biology graphical notation (SBGN [23]).

Exploring other parameter sets from Table 2

In the main article parameter set #6 (Table 2) was chosen because it reproduced (without being forced) correct values for a) total body iron, b) transferrin saturation, and c) RBC half-life. This model was then further shown to not adequately explain the experimental data of Schüman *et al.* [20] for high-iron diet. Questions arose whether the other parameter sets of Table 2 could be able to explain the high-iron diet data. We tested this by attempting to fit the high-iron diet data with each one of those data sets (by adjusting only the parameters vDiet and synthesis rate of hepcidin). None of the other data sets had a better fit to the data than #6 as is displayed in Figure S3 below; particularly all of the models showed an accumulation of iron in RBC larger than observed (Fig. S3D), and a lower accumulation of iron in the liver than observed (Fig S3E). Thus none of the independent parameter fits obtained were able to explain the high iron diet observations. This strengthens the suggestion that further regulatory

interactions are required to explain that data set; and weakens the possibility that such failure is due to inadequate parameter estimates.

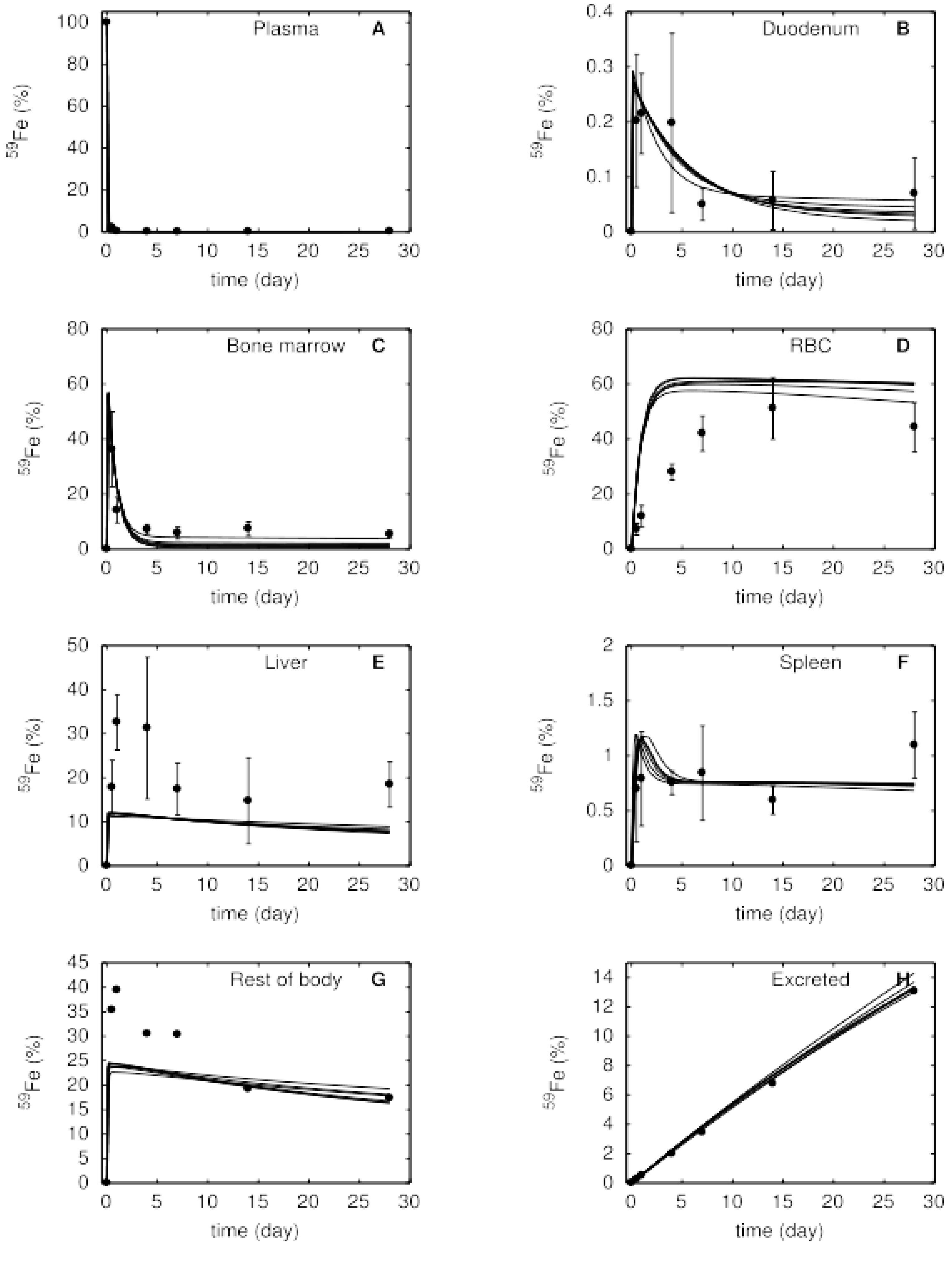

Figure S3. Model simulations of the iron-rich diet data set in Schüman *et al.* [20] carried out with the models of all parameter sets from Table 2. Continuous lines are from each of the model parameter sets, while filled circles represent the data from [20] (vertical bars represent one standard deviation). The

abscissa represents the proportion of total injected ^{59}Fe, the ordinate is time after injection. **A** plasma, **B** duodenum, **C** bone marrow, **D** red blood cells, **E** liver, **F** spleen, **G** rest of body, and **H** excreted. Note that the standard deviation in panel **A** is smaller than the size of the symbols, while in panels **G** and **H** it was not indicated as it is very large because these data are algebraic sums of various terms.

Fitting the model to all diet data sets together

Here we attempted to see if combining all three data sets from Schüman *et al.* [20] for parameter estimation could result in a model that provide an adequate reproduction of all the data. The results from the best model obtained are depicted in Figs. S4-S6. It is clear that even in this situation it was not possible to parameterize the model such that it could represent all data sets well. Note that even in this case, for iron-rich diet, the model accumulates more iron in RBC (Fig. S6D) and less in the liver (Fig. S6E) than in the experimental observations.

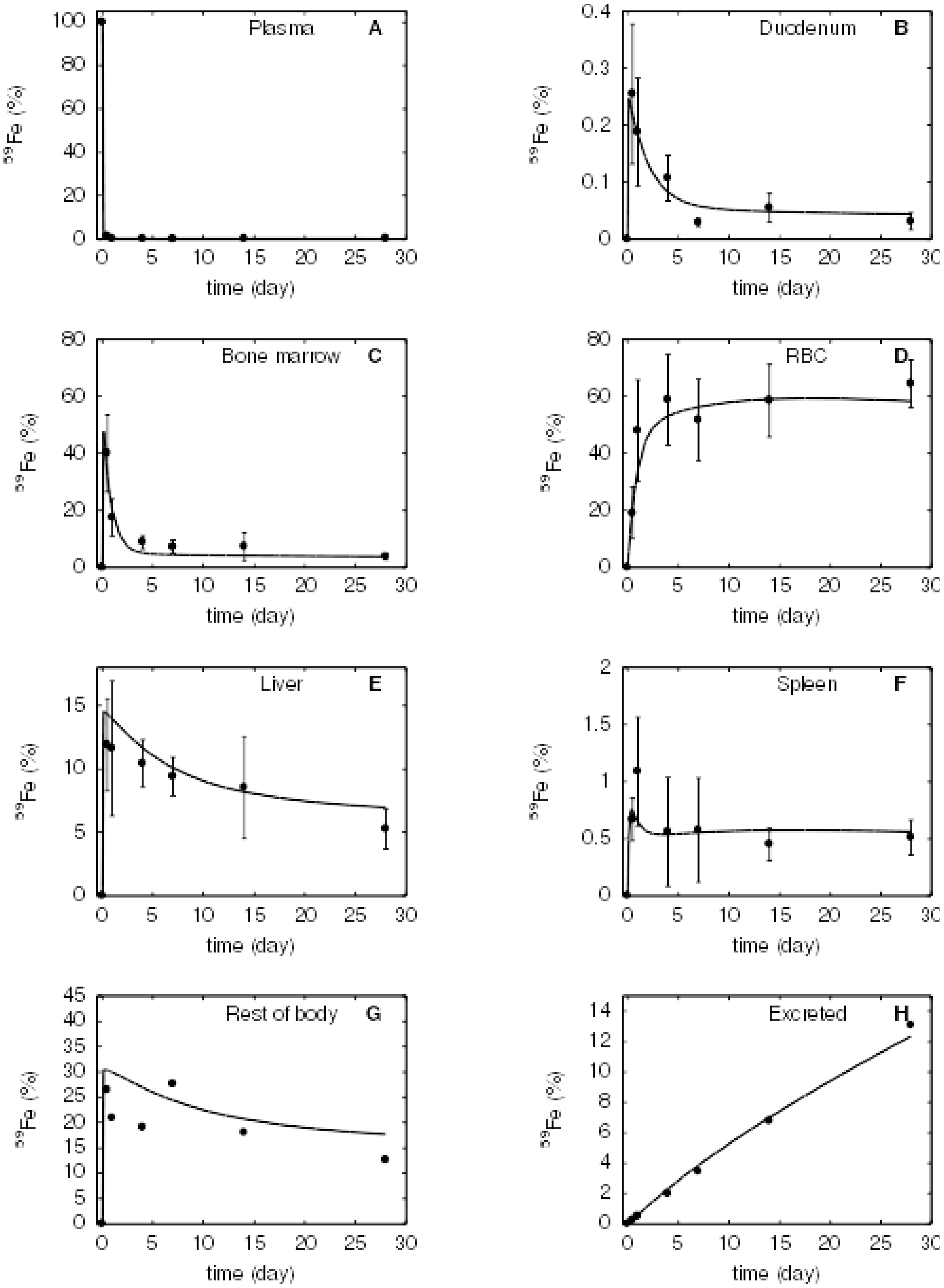

Figure S4. Simulation of the model calibrated with the three data sets against the experimental data of of the adequate iron data set in Schüman *et al.* [20]. Continuous lines are the model simulation, while filled circles represent the data from [20] (vertical bars represent one standard deviation). The abscissa represents the proportion of total injected ^{59}Fe, the ordinate is time after injection. **A** plasma, **B** duodenum, **C** bone marrow, **D** red blood cells, **E** liver, **F** spleen, **G** rest of body, and **H** excreted. Note that the standard deviation in panel **A** is smaller than the size of the symbols, while in panels **G** and **H** it was not indicated as it is very large because these data are algebraic sums of various terms.

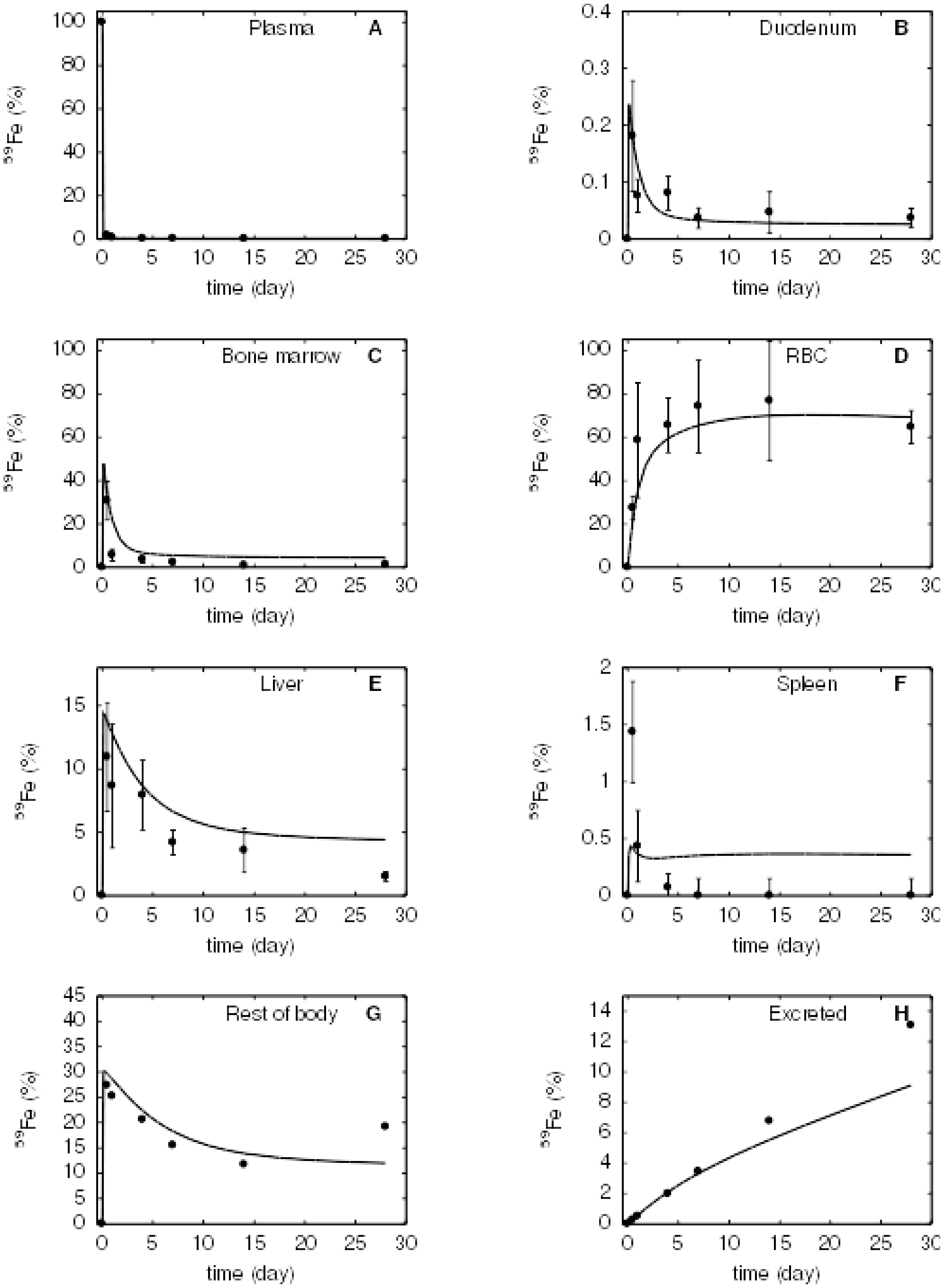

Figure S5. Simulation of the model calibrated with the three data sets against the experimental data of of the iron-deficient data set in Schüman *et al.* [20]. Continuous lines are the model simulation, while filled circles represent the data from [20] (vertical bars represent one standard deviation). The abscissa represents the proportion of total injected ^{59}Fe, the ordinate is time after injection. **A** plasma, **B** duodenum, **C** bone marrow, **D** red blood cells, **E** liver, **F** spleen, **G** rest of body, and **H** excreted. Note that the standard deviation in panel **A** is smaller than the size of the symbols, while in panels **G** and **H** it was not indicated as it is very large because these data are algebraic sums of various terms.

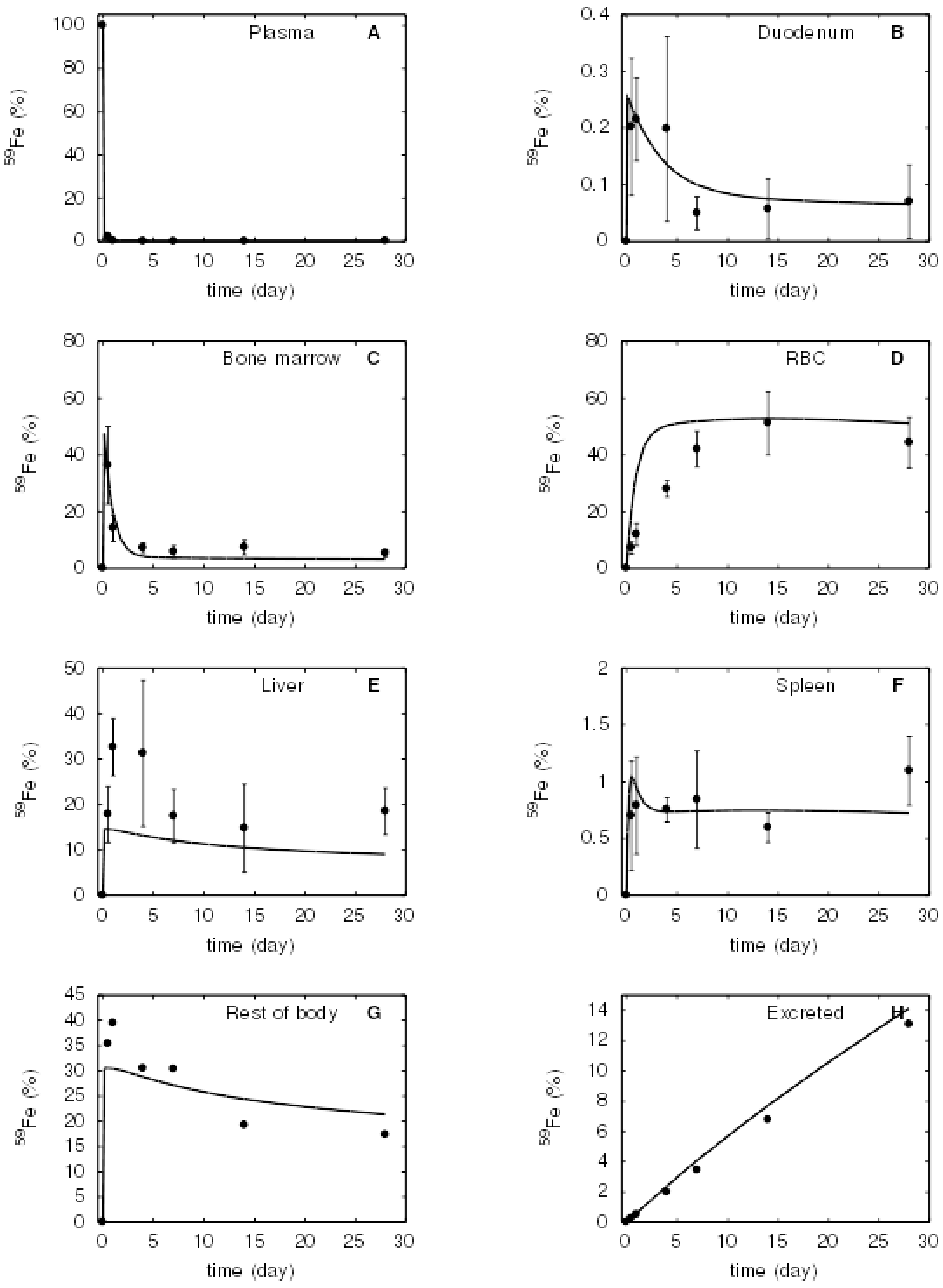

Figure S6. Simulation of the model calibrated with the three data sets against the experimental data of of the iron-rich data set in Schüman *et al.* [20]. Continuous lines are the model simulation, while filled circles represent the data from [20] (vertical bars represent one standard deviation). The abscissa represents the proportion of total injected ^{59}Fe, the ordinate is time after injection. **A** plasma, **B** duodenum, **C** bone marrow, **D** red blood cells, **E** liver, **F** spleen, **G** rest of body, and **H** excreted. Note that the standard deviation in panel **A** is smaller than the size of the symbols, while in panels **G** and **H** it was not indicated as it is very large because these data are algebraic sums of various terms.

Fitting the model to the iron-rich diet

Finally we attempted to see if fitting the model only to the iron-rich diet data set from Schüman *et al.* [20] would produce a model that could be validated by the adequate and deficient iron diet data sets from Schüman *et al.* [20]. The results from the 8 independent fits are depicted in Figs. S7-S8, and the parameter values for each one are listed in Table S1. It is clear that none of the fits of to the rich data can be validated by the two other experimental data sets. This confirms that the current model, where only hepcidin regulates iron mobility, does not adequately describe the physiology across different dietary iron regimes.

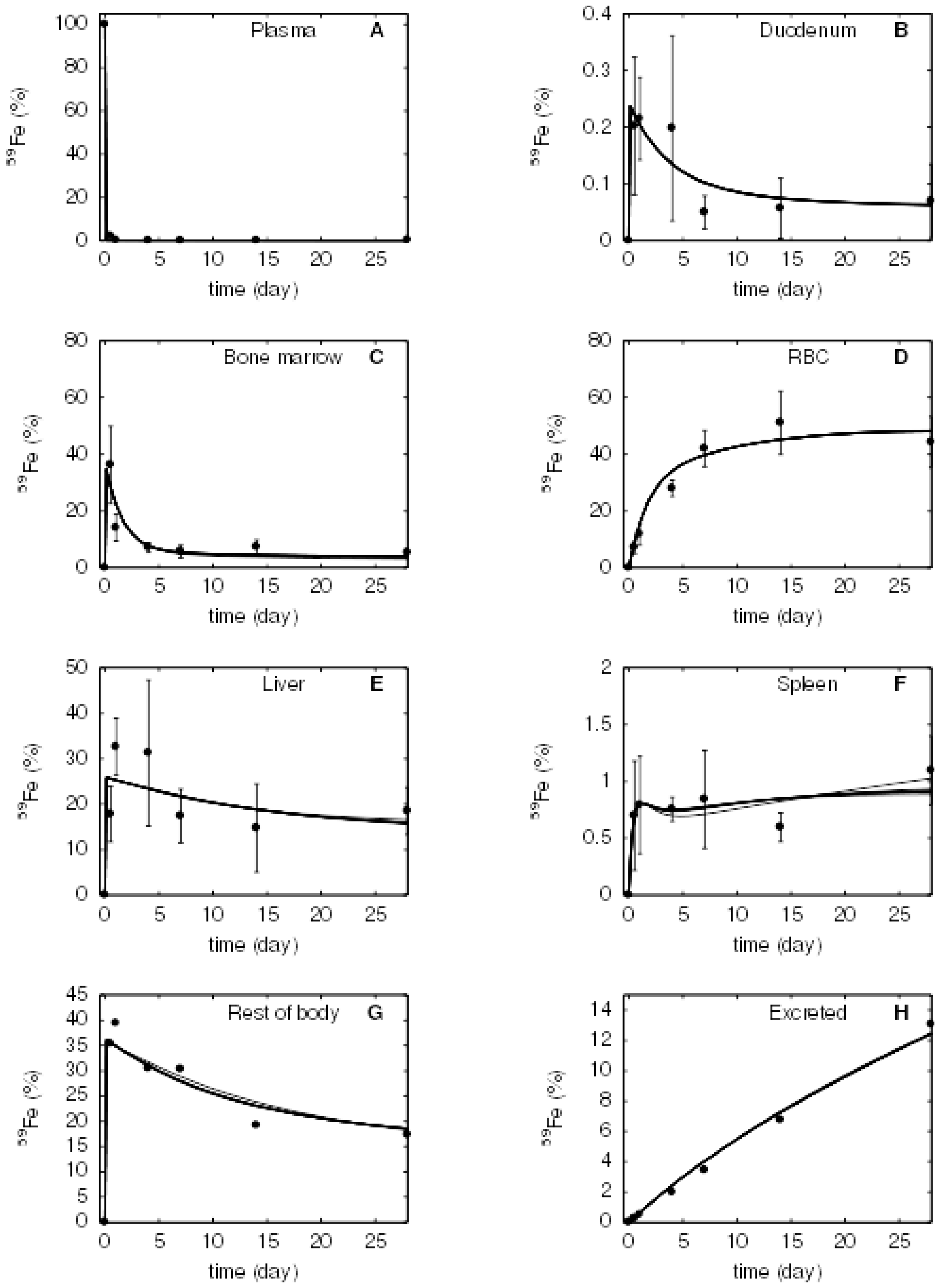

Figure S7. Independent model fits calibrated with the iron-rich diet data in Schüman *et al.* [20] against the (calibration) iron-rich diet data from Schüman *et al.* [20]. Continuous lines are the model

simulation, while filled circles represent the data from [20] (vertical bars represent one standard deviation). The abscissa represents the proportion of total injected ^{59}Fe, the ordinate is time after injection. **A** plasma, **B** duodenum, **C** bone marrow, **D** red blood cells, **E** liver, **F** spleen, **G** rest of body, and **H** excreted. Note that the standard deviation in panel **A** is smaller than the size of the symbols, while in panels **G** and **H** it was not indicated as it is very large because these data are algebraic sums of various terms.

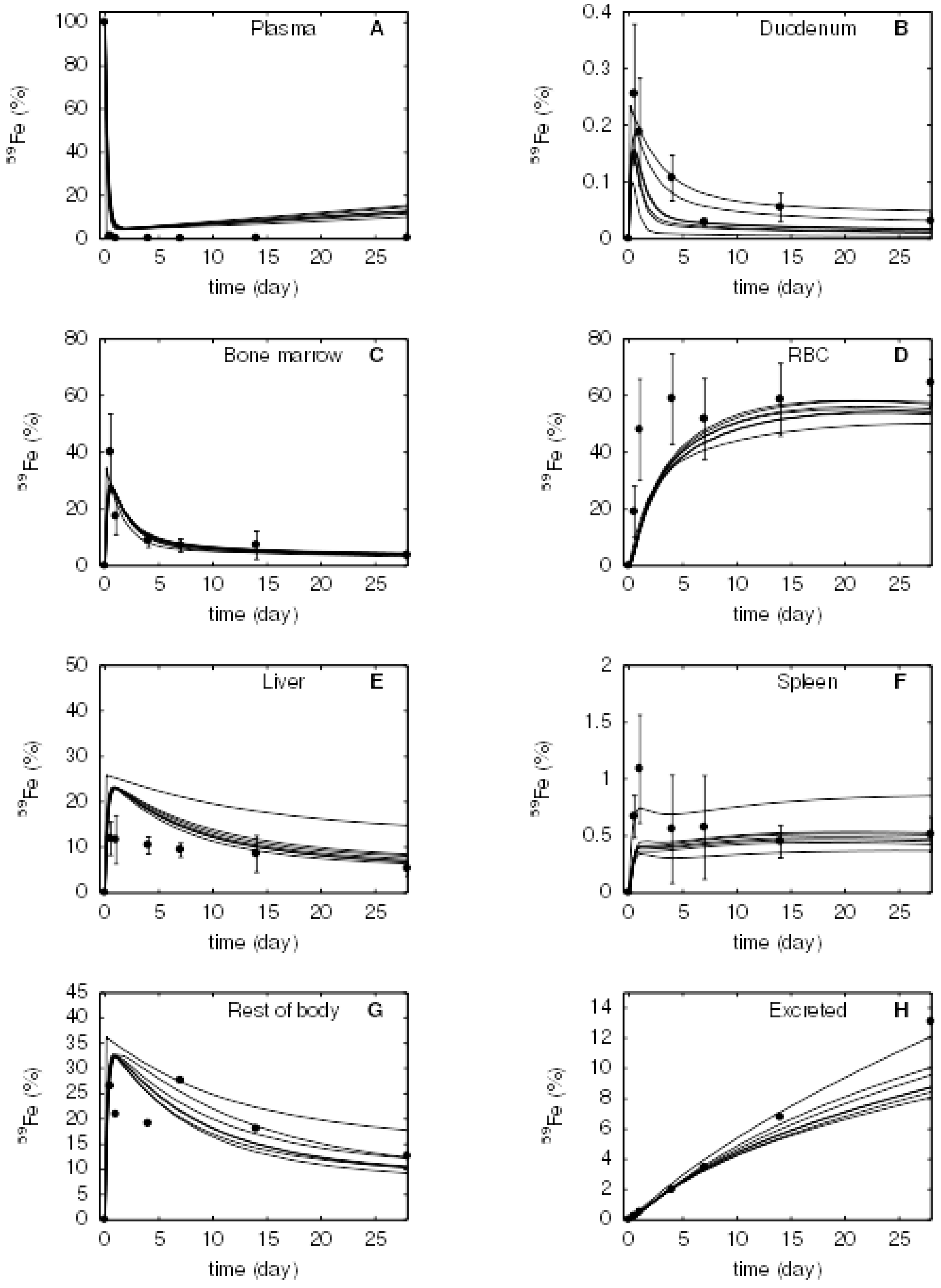

Figure S8. Simulation of the model calibrated with the iron-rich diet data set against the experimental data of of the adequate iron diet data set in Schüman *et al.* [20]. Continuous lines are the model simulation, while filled circles represent the data from [20] (vertical bars represent one standard deviation). The abscissa represents the proportion of total injected ^{59}Fe, the ordinate is time after

injection. **A** plasma, **B** duodenum, **C** bone marrow, **D** red blood cells, **E** liver, **F** spleen, **G** rest of body, and **H** excreted. Note that the standard deviation in panel **A** is smaller than the size of the symbols, while in panels **G** and **H** it was not indicated as it is very large because these data are algebraic sums of various terms.

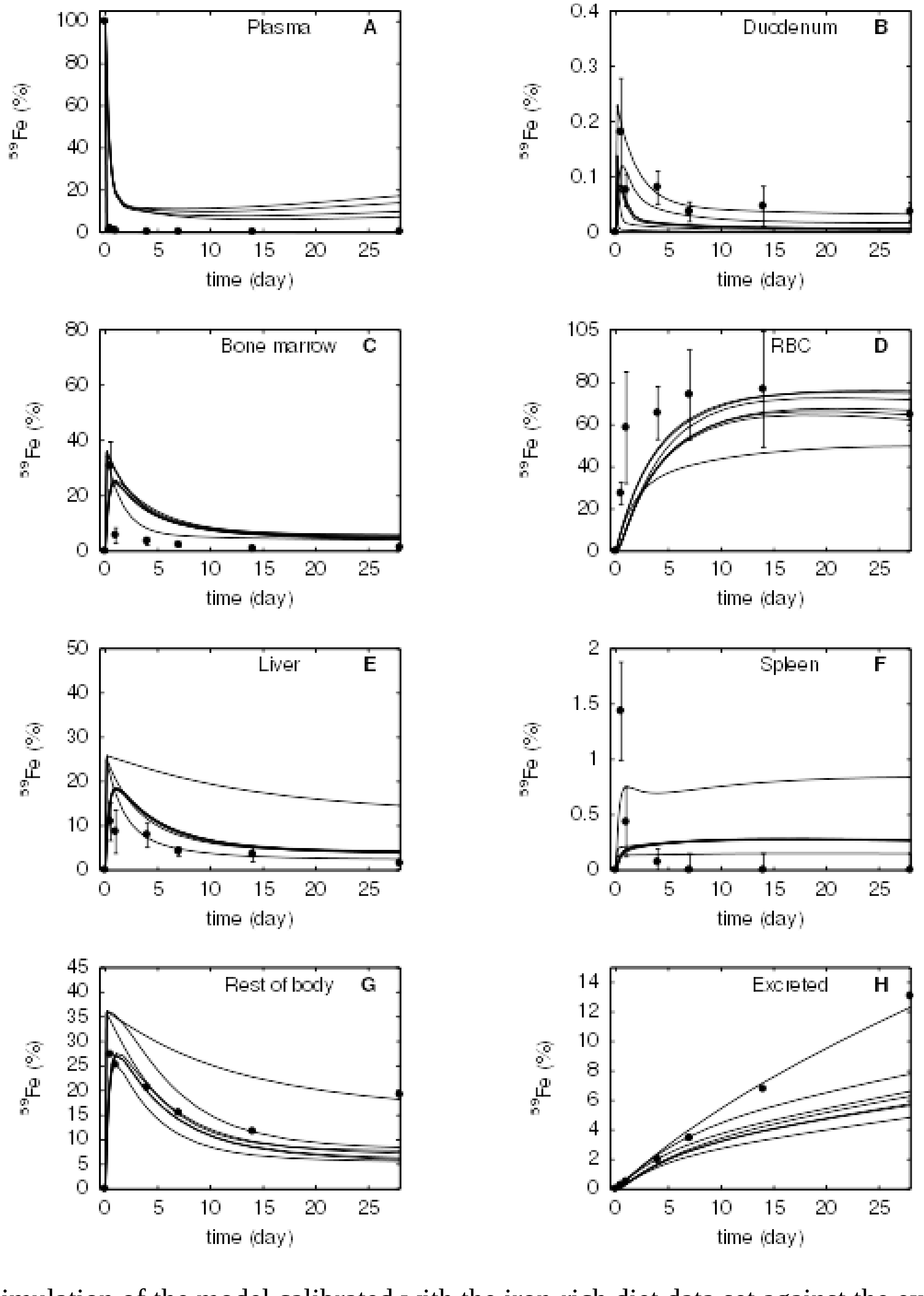

Figure S9. Simulation of the model calibrated with the iron-rich diet data set against the experimental data of of the iron-deficient diet data set in Schüman *et al.* [20]. Continuous lines are the model simulation, while filled circles represent the data from [20] (vertical bars represent one standard deviation). The abscissa represents the proportion of total injected ^{59}Fe, the ordinate is time after injection. **A** plasma, **B** duodenum, **C** bone marrow, **D** red blood cells, **E** liver, **F** spleen, **G** rest of body, and **H** excreted. Note that the standard deviation in panel **A** is smaller than the size of the symbols,

while in panels **G** and **H** it was not indicated as it is very large because these data are algebraic sums of various terms.

Table S1 – Independent parameter estimates and properties of models calibrated against the high iron diet data set.

	**#1**	**#2**	**#3**	**#4**	**#5**	**#6**	**#7**	**#8**
Optimization algorithms[*]	SA	PS Praxis	SRES HJ	SRES	SRES	SRES	SRES Praxis	PS
Sum of squares res.	0.035	0.03516	0.0353	0.03541	0.0355	0.03552	0.0357	0.03571
kInDuo (d^{-1})	1.02	0.719	0.38	0.158	0.765	0.298	0.435	0.144
kInLiver (d^{-1})	109	77.7	40.8	17	82.1	32.1	46.7	15.5
kInRBC (d^{-1})	0.559	0.54	0.567	0.571	0.565	0.568	0.568	0.577
kInRest (d^{-1})	150	106	56.9	23.8	115	44.9	65.6	21.8
kInBM (d^{-1})	155	110	58.3	24.2	117	45.6	66.6	22.1
kDuoLoss (d^{-1})	0.0015	0.00149	0.00133	0.000814	0.00117	0.000807	0.000502	0.000501
kRestOut (d^{-1})	0.0181	0.0179	0.0181	0.0182	0.0181	0.0182	0.0182	0.0182
kBMSpleen (d^{-1})	0.0836	0.073	0.0779	0.0775	0.0764	0.0765	0.0754	0.0772
VDuoNTBI (mol d^{-1})	33.3	23.7	5	0.893	23.8	3.65	13.3	7.36
VLiverNTBI (mol d^{-1})	2.64	1.58	0.642	0.15	4.02	0.679	3.92	3.2
VSpleenNTBI (mol d^{-1})	62.6	38.9	14.8	3.5	92.2	15.7	89.9	74.4
VRestNTBI (mol d^{-1})	1.23	0.513	0.449	0.106	3.2	0.53	3.71	3.24
vRBCSpleen (d^{-1})	0.0439	0.0356	0.0457	0.0465	0.046	0.0463	0.0466	0.0482
Km (M)	0.0436	0.0194	0.047	0.021	0.149	0.0549	0.338	0.776
vDiet (M d^{-1})	0.15	0.109	0.0551	0.0173	0.101	0.0335	0.0602	0.0134
kNTBI_Fe1Tf ($M^{-1}d^{-1}$)	$1.08.10^9$	$1.08.10^9$	$1.08.10^9$	$1.08.10^9$	$1.08.10^9$	$1.08.10^9$	$1.08.10^9$	$1.08.10^9$
kFe1Tf_Fe2Tf ($M^{-1}d^{-1}$)	$1.08.10^9$	$1.08.10^9$	$1.08.10^9$	$1.08.10^9$	$1.08.10^9$	$1.08.10^9$	$1.08.10^9$	$1.08.10^9$
TF saturation (%)	62.5	82.7	57.6	74.8	62.9	73.8	57.6	69.9
Total Fe (mg)	12.3	14.2	4.64	2.85	9.29	4.64	5.21	2.56
ksHepcidin	$1.59.10^{-7}$	$1.65.10^{-7}$	$5.52.10^{-7}$	$2.83.10^{-8}$	$1.25.10^{-7}$	$5.55.10^{-8}$	$6.42.10^{-8}$	$2.33.10^{-8}$
RBC trans. Time (d)	22.8	28.1	21.9	21.5	21.8	21.6	21.5	20.7

* Key to optimization algorithms: SA – simulated annealing; PS – particle swarm; SRES - evolution strategy with stochastic ranking; HJ – Hooke and Jeeves

In order to assess the similarity of each parameter value between the models calibrated with the adequate iron diet data (Table 2, main article) and the models calibrated with the iron-rich diet data (Table S1), we computed the two-tailed Welch's *t*-test, which estimates the probability that the mean values in each table (for corresponding parameters, *i.e.* rows) are equal (the null hypothesis). Table S2 lists the corresponding *p*-values obtained for each null hypothesis.

Table S2 – Comparison of parameter values from the models calibrated to adequate iron diet data (Table 2) and those calibrated against iron-rich diet data (Table S1) carried out with a *t*-test. Parameters are listed in increasing order of the *p*-value.

**Parameter**	***p*-value**
kRestOut	$3.85 \cdot 10^{-15}$
kInRBC	$7.50 \cdot 10^{-11}$
kInLiver	0.0228
vDiet	0.0243
kInRest	0.0310
VRestNTBI	0.0736
kInDuo	0.0860
VLiverNTBI	0.0906
VDuoNTBI	0.101
kBMSpleen	0.144
kDuoLoss	0.145
kInBM	0.239
VSpleenNTBI	0.580
Km	0.901
vRBCSpleen	0.949

Model Equations

Below is the full mathematical model as created by the COPASI software based on the input of a) reaction stoichiometry and b) kinetic rate laws for each reaction. Included are also algebraic equations used to calculate several quantities of interest. $\mathcal{N}$ represents the Avogadro number; "$\mathrm{Injected}$" represents the total injected radioactive iron (in particle numbers). The subscript $_{\mathrm{PN}}$ indicates that the corresponding variable is the number of particles rather than concentration (*e.g.* $\mathrm{Tf}_{\mathrm{PN}}$ for the number of particles of Tf, and $[\mathrm{Tf}]$ for its concentration).

$$
\begin{aligned}
\frac{\mathrm{d}\big([\mathrm{FeDuo}^{*}]\cdot V_{\mathrm{Duodenum}}\big)}{\mathrm{d}t} &= -\left(\frac{\mathrm{VDuoNTBI}\cdot V_{\mathrm{Duodenum}}\cdot[\mathrm{FeDuo}^{*}]}{(\mathrm{Km}+[\mathrm{FeDuo}^{*}]+[\mathrm{FeDuo}])\cdot\left(1+\frac{[\mathrm{Hepcidin}]}{\mathrm{Ki}}\right)}\right)\\
&\quad +2\cdot(\mathrm{kInDuo}\cdot[\mathrm{Fe2Tf}^{**}]\cdot V_{\mathrm{Plasma}})\\
&\quad +(\mathrm{kInDuo}\cdot[\mathrm{Fe1Tf}^{**}]\cdot V_{\mathrm{Plasma}})\\
&\quad +(\mathrm{kInDuo}\cdot[\mathrm{Fe2Tf}^{**}]\cdot V_{\mathrm{Plasma}})\\
&\quad -(\mathrm{kDuoLoss}\cdot[\mathrm{FeDuo}^{*}]\cdot V_{\mathrm{Duodenum}})\\
\frac{\mathrm{d}([\mathrm{FeDuo}]\cdot V_{\mathrm{Duodenum}})}{\mathrm{d}t} &= +V_{\mathrm{Duodenum}}\cdot(\mathrm{vDiet})\\
&\quad -\left(\frac{\mathrm{VDuoNTBI}\cdot V_{\mathrm{Duodenum}}\cdot[\mathrm{FeDuo}]}{(\mathrm{Km}+[\mathrm{FeDuo}]+[\mathrm{FeDuo}^{*}])\cdot\left(1+\frac{[\mathrm{Hepcidin}]}{\mathrm{Ki}}\right)}\right)\\
&\quad -(\mathrm{kDuoLoss}\cdot[\mathrm{FeDuo}]\cdot V_{\mathrm{Duodenum}})\\
&\quad +2\cdot(\mathrm{kInDuo}\cdot[\mathrm{Fe2Tf}]\cdot V_{\mathrm{Plasma}})\\
&\quad +(\mathrm{kInDuo}\cdot[\mathrm{Fe1Tf}]\cdot V_{\mathrm{Plasma}})\\
&\quad +(\mathrm{kInDuo}\cdot[\mathrm{Fe2Tf}^{**}]\cdot V_{\mathrm{Plasma}})\\
\frac{\mathrm{d}\big([\mathrm{FeBM}^{*}]\cdot V_{\mathrm{BoneMarrow}}\big)}{\mathrm{d}t} &= +2\cdot(\mathrm{kInBM}\cdot[\mathrm{Fe2Tf}^{**}]\cdot V_{\mathrm{Plasma}})\\
&\quad +(\mathrm{kInBM}\cdot[\mathrm{Fe1Tf}^{**}]\cdot V_{\mathrm{Plasma}})\\
&\quad +(\mathrm{kInBM}\cdot[\mathrm{Fe2Tf}^{**}]\cdot V_{\mathrm{Plasma}})\\
&\quad -(\mathrm{kInRBC}\cdot[\mathrm{FeBM}^{*}]\cdot V_{\mathrm{BoneMarrow}})\\
&\quad -(\mathrm{kBMSpleen}\cdot[\mathrm{FeBM}^{*}]\cdot V_{\mathrm{BoneMarrow}})\\
\frac{\mathrm{d}([\mathrm{FeBM}]\cdot V_{\mathrm{BoneMarrow}})}{\mathrm{d}t} &= +2\cdot(\mathrm{kInBM}\cdot[\mathrm{Fe2Tf}]\cdot V_{\mathrm{Plasma}})\\
&\quad +(\mathrm{kInBM}\cdot[\mathrm{Fe1Tf}]\cdot V_{\mathrm{Plasma}})\\
&\quad +(\mathrm{kInBM}\cdot[\mathrm{Fe2Tf}^{**}]\cdot V_{\mathrm{Plasma}})\\
&\quad -(\mathrm{kInRBC}\cdot[\mathrm{FeBM}]\cdot V_{\mathrm{BoneMarrow}})\\
&\quad -(\mathrm{kBMSpleen}\cdot[\mathrm{FeBM}]\cdot V_{\mathrm{BoneMarrow}})\\
\frac{\mathrm{d}\big([\mathrm{FeRBC}^{*}]\cdot V_{\mathrm{RBC}}\big)}{\mathrm{d}t} &= -(\mathrm{vRBCSpleen}\cdot[\mathrm{FeRBC}^{*}]\cdot V_{\mathrm{RBC}})\\
&\quad +(\mathrm{kInRBC}\cdot[\mathrm{FeBM}^{*}]\cdot V_{\mathrm{BoneMarrow}})\\
\frac{\mathrm{d}([\mathrm{FeRBC}]\cdot V_{\mathrm{RBC}})}{\mathrm{d}t} &= -(\mathrm{vRBCSpleen}\cdot[\mathrm{FeRBC}]\cdot V_{\mathrm{RBC}})\\
&\quad +(\mathrm{kInRBC}\cdot[\mathrm{FeBM}]\cdot V_{\mathrm{BoneMarrow}})
\end{aligned}
$$

$$\frac{\mathrm{d}\big([\text{FeSpleen}^*] \cdot V_{\text{Spleen}}\big)}{\mathrm{d}t} = + (\text{vRBCSpleen} \cdot [\text{FeRBC}^*] \cdot V_{\text{RBC}})$$

$$- \left(\frac{\text{VSpleenNTBI} \cdot V_{\text{Spleen}} \cdot [\text{FeSpleen}^*]}{(\text{Km} + [\text{FeSpleen}^*] + [\text{FeSpleen}]) \cdot \left(1 + \frac{[\text{Hepcidin}]}{\text{Ki}}\right)} \right)$$

$$+ (\text{kBMSpleen} \cdot [\text{FeBM}^*] \cdot V_{\text{BoneMarrow}})$$

$$\frac{\mathrm{d}\big([\text{FeSpleen}] \cdot V_{\text{Spleen}}\big)}{\mathrm{d}t} = + (\text{vRBCSpleen} \cdot [\text{FeRBC}] \cdot V_{\text{RBC}})$$

$$- \left(\frac{\text{VSpleenNTBI} \cdot V_{\text{Spleen}} \cdot [\text{FeSpleen}]}{(\text{Km} + [\text{FeSpleen}] + [\text{FeSpleen}^*]) \cdot \left(1 + \frac{[\text{Hepcidin}]}{\text{Ki}}\right)} \right)$$

$$+ (\text{kBMSpleen} \cdot [\text{FeBM}] \cdot V_{\text{BoneMarrow}})$$

$$\frac{\mathrm{d}\big([\text{FeLiver}^*] \cdot V_{\text{Liver}}\big)}{\mathrm{d}t} = + (\text{kInLiver} \cdot [\text{Fe1Tf}^{**}] \cdot V_{\text{Plasma}})$$

$$- \left(\frac{\text{VLiverNTBI} \cdot V_{\text{Liver}} \cdot [\text{FeLiver}^*]}{(\text{Km} + [\text{FeLiver}^*] + [\text{FeLiver}]) \cdot \left(1 + \frac{[\text{Hepcidin}]}{\text{Ki}}\right)} \right)$$

$$+ (\text{kInLiver} \cdot [\text{Fe2Tf}^{**}] \cdot V_{\text{Plasma}})$$

$$+ 2 \cdot (\text{kInLiver} \cdot [\text{Fe2Tf}^{**}] \cdot V_{\text{Plasma}})$$

$$\frac{\mathrm{d}([\text{FeLiver}] \cdot V_{\text{Liver}})}{\mathrm{d}t} = - \left(\frac{\text{VLiverNTBI} \cdot V_{\text{Liver}} \cdot [\text{FeLiver}]}{(\text{Km} + [\text{FeLiver}] + [\text{FeLiver}^*]) \cdot \left(1 + \frac{[\text{Hepcidin}]}{\text{Ki}}\right)} \right)$$

$$+ 2 \cdot (\text{kInLiver} \cdot [\text{Fe2Tf}] \cdot V_{\text{Plasma}})$$

$$+ (\text{kInLiver} \cdot [\text{Fe1Tf}] \cdot V_{\text{Plasma}})$$

$$+ (\text{kInLiver} \cdot [\text{Fe2Tf}^{**}] \cdot V_{\text{Plasma}})$$

$$\frac{\mathrm{d}\big([\text{FeRest}^*] \cdot V_{\text{RestOfBody}}\big)}{\mathrm{d}t} = - \left(\frac{\text{VRestNTBI} \cdot V_{\text{RestOfBody}} \cdot [\text{FeRest}^*]}{(\text{Km} + [\text{FeRest}^*] + [\text{FeRest}]) \cdot \left(1 + \frac{[\text{Hepcidin}]}{\text{Ki}}\right)} \right)$$

$$+ (\text{kInRest} \cdot [\text{Fe1Tf}^{**}] \cdot V_{\text{Plasma}})$$

$$+ (\text{kInRest} \cdot [\text{Fe2Tf}^{**}] \cdot V_{\text{Plasma}})$$

$$+ 2 \cdot (\text{kInRest} \cdot [\text{Fe2Tf}^{**}] \cdot V_{\text{Plasma}})$$

$$- V_{\text{RestOfBody}} \cdot (\text{kRestLoss} \cdot [\text{FeRest}^*])$$

$$\frac{\mathrm{d}\big([\text{FeRest}] \cdot V_{\text{RestOfBody}}\big)}{\mathrm{d}t} = + 2 \cdot (\text{kInRest} \cdot [\text{Fe2Tf}] \cdot V_{\text{Plasma}})$$

$$- V_{\text{RestOfBody}} \cdot (\text{kRestLoss} \cdot [\text{FeRest}])$$

$$- \left(\frac{\text{VRestNTBI} \cdot V_{\text{RestOfBody}} \cdot [\text{FeRest}]}{(\text{Km} + [\text{FeRest}] + [\text{FeRest}^*]) \cdot \left(1 + \frac{[\text{Hepcidin}]}{\text{Ki}}\right)} \right)$$

$$+ (\text{kInRest} \cdot [\text{Fe1Tf}] \cdot V_{\text{Plasma}})$$

$$+ (\text{kInRest} \cdot [\text{Fe2Tf}^{**}] \cdot V_{\text{Plasma}})$$

$$\frac{\mathrm{d}\left([\mathrm{FeOutside}^{*}] \cdot V_{\mathrm{RestOfBody}}\right)}{\mathrm{d}t} = + \left(\mathrm{kDuoLoss} \cdot [\mathrm{FeDuo}^{*}] \cdot V_{\mathrm{Duodenum}}\right) + V_{\mathrm{RestOfBody}} \cdot \left(\mathrm{kRestLoss} \cdot [\mathrm{FeRest}^{*}]\right)$$

$$\frac{\mathrm{d}\left([\mathrm{Hepcidin}] \cdot V_{\mathrm{Plasma}}\right)}{\mathrm{d}t} = + V_{\mathrm{Plasma}} \cdot \mathrm{vHepcidinSynthesis} - V_{\mathrm{Plasma}} \cdot \left(\mathrm{kHepcidinDecay} \cdot [\mathrm{Hepcidin}]\right)$$

$$\begin{aligned}\frac{\mathrm{d}\left([\mathrm{NTBI}^{*}] \cdot V_{\mathrm{Plasma}}\right)}{\mathrm{d}t} = & + \left(\frac{\mathrm{VDuoNTBI} \cdot V_{\mathrm{Duodenum}} \cdot [\mathrm{FeDuo}^{*}]}{(\mathrm{Km} + [\mathrm{FeDuo}^{*}] + [\mathrm{FeDuo}]) \cdot \left(1 + \frac{[\mathrm{Hepcidin}]}{\mathrm{Ki}}\right)}\right) \\ & + \left(\frac{\mathrm{VRestNTBI} \cdot V_{\mathrm{RestOfBody}} \cdot [\mathrm{FeRest}^{*}]}{(\mathrm{Km} + [\mathrm{FeRest}^{*}] + [\mathrm{FeRest}]) \cdot \left(1 + \frac{[\mathrm{Hepcidin}]}{\mathrm{Ki}}\right)}\right) \\ & - V_{\mathrm{Plasma}} \cdot \left(\mathrm{kFe1Tf\_Fe2Tf} \cdot [\mathrm{Fe1Tf}^{**}] \cdot [\mathrm{NTBI}^{*}]\right) \\ & + \left(\frac{\mathrm{VSpleenNTBI} \cdot V_{\mathrm{Spleen}} \cdot [\mathrm{FeSpleen}^{*}]}{(\mathrm{Km} + [\mathrm{FeSpleen}^{*}] + [\mathrm{FeSpleen}]) \cdot \left(1 + \frac{[\mathrm{Hepcidin}]}{\mathrm{Ki}}\right)}\right) \\ & - V_{\mathrm{Plasma}} \cdot \left(\mathrm{kFe1Tf\_Fe2Tf} \cdot [\mathrm{Fe1Tf}] \cdot [\mathrm{NTBI}^{*}]\right) \\ & + \left(\frac{\mathrm{VLiverNTBI} \cdot V_{\mathrm{Liver}} \cdot [\mathrm{FeLiver}^{*}]}{(\mathrm{Km} + [\mathrm{FeLiver}^{*}] + [\mathrm{FeLiver}]) \cdot \left(1 + \frac{[\mathrm{Hepcidin}]}{\mathrm{Ki}}\right)}\right) \\ & - V_{\mathrm{Plasma}} \cdot \left(\mathrm{kNTBI\_Fe1Tf} \cdot [\mathrm{NTBI}^{*}] \cdot [\mathrm{Tf}]\right)\end{aligned}$$

$$\begin{aligned}\frac{\mathrm{d}\left([\mathrm{NTBI}] \cdot V_{\mathrm{Plasma}}\right)}{\mathrm{d}t} = & + \left(\frac{\mathrm{VDuoNTBI} \cdot V_{\mathrm{Duodenum}} \cdot [\mathrm{FeDuo}]}{(\mathrm{Km} + [\mathrm{FeDuo}] + [\mathrm{FeDuo}^{*}]) \cdot \left(1 + \frac{[\mathrm{Hepcidin}]}{\mathrm{Ki}}\right)}\right) \\ & + \left(\frac{\mathrm{VSpleenNTBI} \cdot V_{\mathrm{Spleen}} \cdot [\mathrm{FeSpleen}]}{(\mathrm{Km} + [\mathrm{FeSpleen}] + [\mathrm{FeSpleen}^{*}]) \cdot \left(1 + \frac{[\mathrm{Hepcidin}]}{\mathrm{Ki}}\right)}\right) \\ & + \left(\frac{\mathrm{VLiverNTBI} \cdot V_{\mathrm{Liver}} \cdot [\mathrm{FeLiver}]}{(\mathrm{Km} + [\mathrm{FeLiver}] + [\mathrm{FeLiver}^{*}]) \cdot \left(1 + \frac{[\mathrm{Hepcidin}]}{\mathrm{Ki}}\right)}\right) \\ & - V_{\mathrm{Plasma}} \cdot \left(\mathrm{kNTBI\_Fe1Tf} \cdot [\mathrm{NTBI}] \cdot [\mathrm{Tf}]\right) \\ & + \left(\frac{\mathrm{VRestNTBI} \cdot V_{\mathrm{RestOfBody}} \cdot [\mathrm{FeRest}]}{(\mathrm{Km} + [\mathrm{FeRest}] + [\mathrm{FeRest}^{*}]) \cdot \left(1 + \frac{[\mathrm{Hepcidin}]}{\mathrm{Ki}}\right)}\right) \\ & - V_{\mathrm{Plasma}} \cdot \left(\mathrm{kFe1Tf\_Fe2Tf} \cdot [\mathrm{Fe1Tf}] \cdot [\mathrm{NTBI}]\right) \\ & - V_{\mathrm{Plasma}} \cdot \left(\mathrm{kFe1Tf\_Fe2Tf} \cdot [\mathrm{Fe1Tf}^{**}] \cdot [\mathrm{NTBI}]\right)\end{aligned}$$

$$\begin{aligned}\frac{\mathrm{d}\left([\mathrm{Fe2Tf}^{**}] \cdot V_{\mathrm{Plasma}}\right)}{\mathrm{d}t} = & - \left(\mathrm{kInBM} \cdot [\mathrm{Fe2Tf}^{**}] \cdot V_{\mathrm{Plasma}}\right) \\ & - \left(\mathrm{kInDuo} \cdot [\mathrm{Fe2Tf}^{**}] \cdot V_{\mathrm{Plasma}}\right) \\ & + V_{\mathrm{Plasma}} \cdot \left(\mathrm{kFe1Tf\_Fe2Tf} \cdot [\mathrm{Fe1Tf}^{**}] \cdot [\mathrm{NTBI}^{*}]\right) \\ & - \left(\mathrm{kInLiver} \cdot [\mathrm{Fe2Tf}^{**}] \cdot V_{\mathrm{Plasma}}\right) \\ & - \left(\mathrm{kInRest} \cdot [\mathrm{Fe2Tf}^{**}] \cdot V_{\mathrm{Plasma}}\right)\end{aligned}$$

$$
\begin{aligned}
\frac{\mathrm{d}\left([\mathrm{Fe2Tf}^{**}]\cdot V_{\mathrm{Plasma}}\right)}{\mathrm{d}t} &= +V_{\mathrm{Plasma}}\cdot(\mathrm{kFe1Tf\_Fe2Tf}\cdot[\mathrm{Fe1Tf}]\cdot[\mathrm{NTBI}^{*}])\\
&+V_{\mathrm{Plasma}}\cdot(\mathrm{kFe1Tf\_Fe2Tf}\cdot[\mathrm{Fe1Tf}^{**}]\cdot[\mathrm{NTBI}])\\
&-(\mathrm{kInDuo}\cdot[\mathrm{Fe2Tf}^{**}]\cdot V_{\mathrm{Plasma}})\\
&-(\mathrm{kInLiver}\cdot[\mathrm{Fe2Tf}^{**}]\cdot V_{\mathrm{Plasma}})\\
&-(\mathrm{kInBM}\cdot[\mathrm{Fe2Tf}^{**}]\cdot V_{\mathrm{Plasma}})\\
&-(\mathrm{kInRest}\cdot[\mathrm{Fe2Tf}^{**}]\cdot V_{\mathrm{Plasma}})
\end{aligned}
$$

$$
\begin{aligned}
\frac{\mathrm{d}([\mathrm{Fe2Tf}]\cdot V_{\mathrm{Plasma}})}{\mathrm{d}t} &= -(\mathrm{kInBM}\cdot[\mathrm{Fe2Tf}]\cdot V_{\mathrm{Plasma}})\\
&-(\mathrm{kInLiver}\cdot[\mathrm{Fe2Tf}]\cdot V_{\mathrm{Plasma}})\\
&-(\mathrm{kInRest}\cdot[\mathrm{Fe2Tf}]\cdot V_{\mathrm{Plasma}})\\
&-(\mathrm{kInDuo}\cdot[\mathrm{Fe2Tf}]\cdot V_{\mathrm{Plasma}})\\
&+V_{\mathrm{Plasma}}\cdot(\mathrm{kFe1Tf\_Fe2Tf}\cdot[\mathrm{Fe1Tf}]\cdot[\mathrm{NTBI}])
\end{aligned}
$$

$$
\begin{aligned}
\frac{\mathrm{d}\left([\mathrm{Fe1Tf}^{**}]\cdot V_{\mathrm{Plasma}}\right)}{\mathrm{d}t} &= -V_{\mathrm{Plasma}}\cdot(\mathrm{kFe1Tf\_Fe2Tf}\cdot[\mathrm{Fe1Tf}^{**}]\cdot[\mathrm{NTBI}^{*}])\\
&-(\mathrm{kInLiver}\cdot[\mathrm{Fe1Tf}^{**}]\cdot V_{\mathrm{Plasma}})\\
&-(\mathrm{kInBM}\cdot[\mathrm{Fe1Tf}^{**}]\cdot V_{\mathrm{Plasma}})\\
&-(\mathrm{kInRest}\cdot[\mathrm{Fe1Tf}^{**}]\cdot V_{\mathrm{Plasma}})\\
&-(\mathrm{kInDuo}\cdot[\mathrm{Fe1Tf}^{**}]\cdot V_{\mathrm{Plasma}})\\
&-V_{\mathrm{Plasma}}\cdot(\mathrm{kFe1Tf\_Fe2Tf}\cdot[\mathrm{Fe1Tf}^{**}]\cdot[\mathrm{NTBI}])\\
&+V_{\mathrm{Plasma}}\cdot(\mathrm{kNTBI\_Fe1Tf}\cdot[\mathrm{NTBI}^{*}]\cdot[\mathrm{Tf}])
\end{aligned}
$$

$$
\begin{aligned}
\frac{\mathrm{d}([\mathrm{Fe1Tf}]\cdot V_{\mathrm{Plasma}})}{\mathrm{d}t} &= +V_{\mathrm{Plasma}}\cdot(\mathrm{kNTBI\_Fe1Tf}\cdot[\mathrm{NTBI}]\cdot[\mathrm{Tf}])\\
&-V_{\mathrm{Plasma}}\cdot(\mathrm{kFe1Tf\_Fe2Tf}\cdot[\mathrm{Fe1Tf}]\cdot[\mathrm{NTBI}])\\
&-(\mathrm{kInLiver}\cdot[\mathrm{Fe1Tf}]\cdot V_{\mathrm{Plasma}})\\
&-(\mathrm{kInBM}\cdot[\mathrm{Fe1Tf}]\cdot V_{\mathrm{Plasma}})\\
&-(\mathrm{kInRest}\cdot[\mathrm{Fe1Tf}]\cdot V_{\mathrm{Plasma}})\\
&-(\mathrm{kInDuo}\cdot[\mathrm{Fe1Tf}]\cdot V_{\mathrm{Plasma}})\\
&-V_{\mathrm{Plasma}}\cdot(\mathrm{kFe1Tf\_Fe2Tf}\cdot[\mathrm{Fe1Tf}]\cdot[\mathrm{NTBI}^{*}])
\end{aligned}
$$

$$\begin{aligned}
\frac{\mathrm{d}([\mathrm{Tf}]\cdot V_{\mathrm{Plasma}})}{\mathrm{d}t} = & + (\mathrm{kInBM}\cdot[\mathrm{Fe2Tf}^{**}]\cdot V_{\mathrm{Plasma}}) \\
& + (\mathrm{kInDuo}\cdot[\mathrm{Fe2Tf}^{**}]\cdot V_{\mathrm{Plasma}}) \\
& + (\mathrm{kInLiver}\cdot[\mathrm{Fe1Tf}^{**}]\cdot V_{\mathrm{Plasma}}) \\
& + (\mathrm{kInBM}\cdot[\mathrm{Fe1Tf}^{**}]\cdot V_{\mathrm{Plasma}}) \\
& + (\mathrm{kInRest}\cdot[\mathrm{Fe1Tf}^{**}]\cdot V_{\mathrm{Plasma}}) \\
& + (\mathrm{kInDuo}\cdot[\mathrm{Fe1Tf}^{**}]\cdot V_{\mathrm{Plasma}}) \\
& + (\mathrm{kInBM}\cdot[\mathrm{Fe2Tf}]\cdot V_{\mathrm{Plasma}}) \\
& - V_{\mathrm{Plasma}}\cdot(\mathrm{kNTBI_Fe1Tf}\cdot[\mathrm{NTBI}]\cdot[\mathrm{Tf}]) \\
& + (\mathrm{kInLiver}\cdot[\mathrm{Fe2Tf}]\cdot V_{\mathrm{Plasma}}) \\
& + (\mathrm{kInRest}\cdot[\mathrm{Fe2Tf}]\cdot V_{\mathrm{Plasma}}) \\
& + (\mathrm{kInDuo}\cdot[\mathrm{Fe2Tf}]\cdot V_{\mathrm{Plasma}}) \\
& + (\mathrm{kInLiver}\cdot[\mathrm{Fe1Tf}]\cdot V_{\mathrm{Plasma}}) \\
& + (\mathrm{kInBM}\cdot[\mathrm{Fe1Tf}]\cdot V_{\mathrm{Plasma}}) \\
& + (\mathrm{kInRest}\cdot[\mathrm{Fe1Tf}]\cdot V_{\mathrm{Plasma}}) \\
& + (\mathrm{kInDuo}\cdot[\mathrm{Fe1Tf}]\cdot V_{\mathrm{Plasma}}) \\
& + (\mathrm{kInDuo}\cdot[\mathrm{Fe2Tf}^{**}]\cdot V_{\mathrm{Plasma}}) \\
& + (\mathrm{kInLiver}\cdot[\mathrm{Fe2Tf}^{**}]\cdot V_{\mathrm{Plasma}}) \\
& + (\mathrm{kInBM}\cdot[\mathrm{Fe2Tf}^{**}]\cdot V_{\mathrm{Plasma}}) \\
& + (\mathrm{kInRest}\cdot[\mathrm{Fe2Tf}^{**}]\cdot V_{\mathrm{Plasma}}) \\
& - V_{\mathrm{Plasma}}\cdot(\mathrm{kNTBI_Fe1Tf}\cdot[\mathrm{NTBI}^{*}]\cdot[\mathrm{Tf}]) \\
& + (\mathrm{kInLiver}\cdot[\mathrm{Fe2Tf}^{**}]\cdot V_{\mathrm{Plasma}}) \\
& + (\mathrm{kInRest}\cdot[\mathrm{Fe2Tf}^{**}]\cdot V_{\mathrm{Plasma}})
\end{aligned}$$

$$\text{Total\_Fe}^* = \text{FeDuo}^*_{\text{PN}} + \text{FeLiver}^*_{\text{PN}} + \text{FeSpleen}^*_{\text{PN}} + \text{FeRBC}^*_{\text{PN}} + \text{FeRest}^*_{\text{PN}} + \text{Fe2Tf}^*_{\text{PN}} + \text{NTBI}^*_{\text{PN}} + 2 \cdot \text{Fe2Tf}^{**}_{\text{PN}} + \text{Fe1Tf}^*_{\text{PN}} + \text{FeBM}^*_{\text{PN}}$$

$$\text{Total\_Fe} = \text{FeDuo}_{\text{PN}} + \text{FeLiver}_{\text{PN}} + \text{FeSpleen}_{\text{PN}} + \text{FeRBC}_{\text{PN}} + \text{FeRest}_{\text{PN}} + \text{Fe2Tf}^*_{\text{PN}} + \text{NTBI}_{\text{PN}} + 2 \cdot \text{Fe2Tf}_{\text{PN}} + \text{Fe1Tf}_{\text{PN}} + \text{FeBM}_{\text{PN}}$$

$$\text{Total\_Fe (conc.)} = \frac{\text{FeDuo}_{\text{PN}} + \text{FeLiver}_{\text{PN}} + \text{FeSpleen}_{\text{PN}} + \text{FeRBC}_{\text{PN}} + \text{FeRest}_{\text{PN}} + 2 \cdot \text{Fe2Tf}_{\text{PN}} + \text{Fe2Tf}^*_{\text{PN}} + \text{Fe1Tf}_{\text{PN}} + \text{NTBI}_{\text{PN}} + \text{FeBM}_{\text{PN}}}{\mathcal{N} \cdot \left(V_{\text{Duodenum}} + V_{\text{Liver}} + V_{\text{Plasma}} + V_{\text{RBC}} + V_{\text{RestOfBody}} + V_{\text{Spleen}}\right)}$$

$$\text{Total\_Fe (g)} = \frac{\text{Total\_Fe} \cdot 55.845}{\mathcal{N}}$$

$$\text{FePlasma} = 2 \cdot \text{Fe2Tf}_{\text{PN}} + \text{Fe2Tf}^*_{\text{PN}} + \text{Fe1Tf}_{\text{PN}} + \text{NTBI}_{\text{PN}}$$

$$\text{FePlasma (conc.)} = \frac{\text{FePlasma}}{V_{\text{Plasma}} \cdot \mathcal{N}}$$

$$\text{FePlasma}^* = 2 \cdot \text{Fe2Tf}^{**}_{\text{PN}} + \text{Fe2Tf}^*_{\text{PN}} + \text{Fe1Tf}^*_{\text{PN}} + \text{NTBI}^*_{\text{PN}}$$

$$\text{FePlasma}^* \text{ (conc.)} = \frac{\text{FePlasma}^*}{V_{\text{Plasma}} \cdot \mathcal{N}}$$

$$\text{Total\_Tf} = \text{Tf}_{\text{PN}} + \text{Fe1Tf}^*_{\text{PN}} + \text{Fe1Tf}_{\text{PN}} + \text{Fe2Tf}^{**}_{\text{PN}} + \text{Fe2Tf}^*_{\text{PN}} + \text{Fe2Tf}_{\text{PN}}$$

$$\text{Total\_Tf (conc).} = \frac{\text{Total\_Tf}}{\mathcal{N} \cdot V_{\text{Plasma}}}$$

$$\text{Total\_Tf mg/ml} = \text{Total\_Tf (conc.)} \cdot 80$$

$$\text{TfSaturation} = \frac{100 \cdot \left(2 \cdot \text{Fe2Tf}^*_{\text{PN}} + 2 \cdot \text{Fe2Tf}_{\text{PN}} + \text{Fe1Tf}^*_{\text{PN}} + \text{Fe1Tf}_{\text{PN}} + 2 \cdot \text{Fe2Tf}^{**}_{\text{PN}}\right)}{2 \cdot \left(\text{Fe2Tf}^*_{\text{PN}} + \text{Fe1Tf}^*_{\text{PN}} + \text{Tf}_{\text{PN}} + \text{Fe2Tf}^{**}_{\text{PN}} + \text{Fe2Tf}_{\text{PN}} + \text{Fe1Tf}_{\text{PN}}\right)}$$

$$\text{PDuo} = \frac{100 \cdot \text{FeDuo}^*_{\text{PN}}}{\text{Injected}}$$

$$\text{PLiver} = \frac{100 \cdot \text{FeLiver}^*_{\text{PN}}}{\text{Injected}}$$

$$\text{PSpleen} = \frac{100 \cdot \text{FeSpleen}^*_{\text{PN}}}{\text{Injected}}$$

$$\text{PRBC} = \frac{100 \cdot \text{FeRBC}^*_{\text{PN}}}{\text{Injected}}$$

$$\text{PRest} = \frac{100 \cdot \text{FeRest}^*_{\text{PN}}}{\text{Injected}}$$

$$\text{PPlasma} = \frac{100 \cdot \left(\text{NTBI}^*_{\text{PN}} + 2 \cdot \text{Fe2Tf}^{**}_{\text{PN}} + \text{Fe2Tf}^*_{\text{PN}} + \text{Fe1Tf}^*_{\text{PN}}\right)}{\text{Injected}}$$

$$\text{POutside} = \frac{100 \cdot \text{FeOutside}^*_{\text{PN}}}{\text{Injected}}$$

$$\text{PBM} = \frac{100 \cdot \text{FeBM}^*_{\text{PN}}}{\text{Injected}}$$

$$\text{Injected} = 3.073 \cdot 10^{15}$$
